# 

**Published:** 1871-08

**Authors:** 


### BOOK NOTICES.

"Thoughts for the Young Men, and for the Young Women of America;" or a few practical words of advice. By L. U. Reavis. With the Ideal Man and the Ideal Woman. By Horace Greeley. Price $1. New York: S. R. Wells, 389 Broadway. The object of this work is to encourage those whose opportunities have not been favorable, and to guide them by honorable purpose and earnest endeavor, to rise above the embarrassments of their condition, and thus call out all that is noble in their nature.

"The Knightly Soldier," being the Biography of Major Henry Ward Camp, Tenth Connecticut Volunteers. Sixth edition. 335 pp. $2. By Chaplain H. Clay Trumbull. A beautiful history of a beautiful Christian, self-sacrificing, heroic life. Noyes, Holmes & Co., publishers, Boston.

"Castles in the Air, and other Phantasies." By BARRY GRAY. "An old man prattling in the sunshine." 1 vol. crown 8vo. Hurd and Houghton, New York: The Riverside Press, Cambridge. $1.75. A volume of sketches in Barry Gray's playful manner. They are not stories crowded with exciting incident, but rambling, good-natured characterizations of people and customs. "Castles in the Air" are the half-reveries of a middle-aged man looking to what might have been.

"The McAllister's," a Temperance Tale. By Mrs. E. T. RICHMOND. Published by the National Temperance Society, 58 Reade Street, New York.

The American Tract Society, Boston, which recently transferred the manufacture and sale of its publications to the Riverside Press, and Hurd and Houghton, has recommenced its issue of good books, and sends out, through its publishers, two books: —

I. "Six Boys: A Mother's Story, as told in Extracts from her Journal." With illustrations. 12mo, cloth, $1.25. This is a story the scene of which is laid in New York and neighborhood, just after the close of the Revolutionary War. A widowed mother is left with six boys on her hands, and her journal gives the growth and change in the lads. Their characters are diverse. A good deal of pains has been taken to give the coloring of the time in manners and customs.

II. "Bible Sketches," Third Series, illustrating the Life of Christ on Earth. Illustrated. 16mo, cloth, $1. The two previous volumes were on the Old Testament. This is independent of them, and gives, in a familiar style for young people, the leading incidents in Christ's life, illustrating them from Eastern customs, and talking about them in the conversational way of one sitting down with a class of youngsters.

Both of these books bear the marks of the careful handling of the Riverside Press, which regards every book worth doing at all as worth doing well. It has given its style to these simple books, and probably will do so even more hereafter, educating the eye as well as the heart of children. The June number of "The Child at Home," from the same source, is a unique specimen of color printing. It is the only paper thus printed in this country, and its bright hues and graceful designs of birds and flowers will make children stretch out their hands for it.

"Great Fortunes and How they were Made." By JAMES D. MCCABE, JR. George Maclean, Nassau Street, New York, publisher. This is an elaborate work of over six hundred pages, well gotten up, illustrative of the early lives of the wealthiest men of our day. Few volumes can be found so full of useful and entertaining knowledge of real life for the old and young.

We have received from Messrs. Lee & Shepard, Boston, "Curiosities of the Law Reporters," by FRANKLIN FISK HEARD; also, "Versatilities," by R. H. NEWELL (Orpheus C. Kerr). These small works are out in attractive style, adapted to a large class of readers, and are certainly very tempting to lovers of instructive books.

"Harper's Monthly Magazine" has attained a circulation of one hundred and thirty thousand copies, each number giving as much reading for thirty-six cents as will be found in a common two-dollar book. Every issue is filled with valuable matter pertaining to fact and fiction, history, news, and general literature, well calculated to instruct, amuse, and elevate.

